# Vertebroplasty and kyphoplasty—evidence-based methods?

**DOI:** 10.3109/17453674.2010.519168

**Published:** 2010-10-08

**Authors:** Ralph Hasserius, Acke Ohlin, Magnus K Karlsson

**Affiliations:** Departments of Clinical Sciences, Lund University and Orthopedics, Skåne University Hospital, Malmö, Sweden

One of the most common fragility fracture and a fracture associated with increased morbidity and mortality is the vertebral compression fracture (Hasserius et al. 2005). The incidence varies, being higher in Scandinavian, American, and Hong Kong Chinese females than in eastern European females. In contrast, the incidence in male Hong Kong Chinese and American Caucasians is lower than in male Europeans (Melton et al. 1993, Lau et al. 1996, O'Neill et al. 1996). The female-to-male ratio in Caucasians is 2:1, with an age-dependent increase in both men and women, giving an incidence of 20 per 10^5^ person-years in individuals below 45 years of age and 1,200 per 10^5^ person-years in those aged 85 years and above (O'Neill et al. 1996).

In light of the enormous costs and the negative health aspects associated with vertebral fractures, percutaneous vertebroplasty and kyphoplasty were introduced. Vertebroplasty by percutaneous injection of bone cement was originally developed in France, and in 1987 it was first reported as one strategy for stabilizing vertebral bodies affected by a tumor (Galibert et al. 1987). In 1989, the method was also shown to stabilize fractured osteoporotic vertebrae (Lapras et al. 1989). Vertebroplasty was hypothetically improved by introduction of the percutaneous balloon kyphoplasty technique (Lieberman et al. 2001). This involves inflating a balloon in the fractured vertebral body in order to restore the anatomy of the fractured vertebra and reduce the fracture-induced kyphosis. The void created is then filled with bone cement. Both methods immediately attracted a great amount of interest. A Medline search in Oct 2004 identified 388 articles on “vertebroplasty” and 92 on “kyphoplasty”. A similar search in Jan 2010 resulted in 1,398 hits for “vertebroplasty” and 1,573 for “kyphoplasty”.

The two techniques, the short-term clinical results, and the possible complications have all been thoroughly reported (Lieberman et al. 2001, Diamond et al. 2003), but up until now there have been no prospective randomized controlled trials (RCTs) published regarding the efficacy of the procedures. Case reports, prospective and retrospective uncontrolled short-term observational studies, and case-control studies have consistently inferred an almost immediate relief of back pain and improved functional status after such procedures in 75–90% of cases (Lieberman et al. 2001, Diamond et al. 2003). These results are based on different estimates of pain relief and have mainly been compared to the preoperative situation but without comparison with controls. Whether or not vertebroplasty or kyphoplasty gives a better outcome than medical treatment is still not known.

Recently, however, several RCTs have improved our understanding (Rauschmann et al. 2004, Gray et al. 2007, Buchbinder et al. 2008, 2009, Kallmes et al. 2009, Wardlaw et al. 2009, Liu et al. 2010). Two multicenter, randomized, double-blind placebo-controlled trials presented in New England Journal of Medicine (NEJM) (Buchbinder et al. 2009, Kallmes et al. 2009)—one study from Australia and one from the USA, of which both denied beneficial effects of vertebroplasty in comparison with medical treatment in patients with osteoporotic vertebral compression fractures—have been debated over the last months. In the Australian study, participants with 1 or 2 painful osteoporotic vertebral fractures confirmed by MRI and with less than 1 year's duration underwent vertebroplasty (n = 35) or a sham procedure (n = 40). Participants were stratified according to duration of symptoms (of less than 6 weeks or 6 weeks or more), and the primary outcome (overall pain) was evaluated after 1 week and at 1, 3, and 6 months. Secondary outcomes were pain at night, pain at rest, physical function, quality of life, and perceived improvement. This report stated that there were substantial reductions in overall pain in both study groups, and that vertebroplasty was not better in any measured outcome at any time compared to the controls. The outcome was the same in individuals with less or more than 6 weeks of pain (Buchbinder et al. 2009).

In the other study, this time from the Mayo Clinic, the researchers randomly assigned 131 patients who had 1 to 3 painful osteoporotic vertebral compression fractures to either vertebroplasty (n = 68) or a simulated procedure without cement (n = 63). The primary outcomes were assessed using the modified Roland-Morris disability questionnaire (RDQ) score and the patient ratings of pain intensity during the 24 hours after the operation and also after 1 month. The patients were allowed to cross over to the other study group after 1 month. This report stated that there was major improvement in the outcome measures in both the vertebroplasty group and the control group and that vertebroplasty did not result in any advantage in measured outcome variables at any time compared to the controls. There was, however, a trend toward beneficial clinical improvement in pain in the vertebroplasty group (64% vs. 48%; p = 0.06) after 1 month and at 3 months. The crossover rate was also higher in the control group than in the vertebroplasty group (43% vs. 12%; p < 0.001) (Kallmes et al. 2009)

A third RCT has supported the 2 NEJM publications, this time involving 50 patients with acute or subacute osteoporotic vertebral fractures (< 2 weeks and between 2 and 8 weeks, respectively) who were randomized to either vertebroplasty or medical treatment. This report stated that reduction in pain was similar in the 2 groups and that there was no statistically significant difference in the other parameters when comparing the results at inclusion and after 3 months within both groups, and virtually none between the groups after 3 months (Rousing et al. 2009). The authors concluded that most patients with acute or subacute painful osteoporotic compression fractures of the spine will spontaneously recover after a few months. This view is opposed by other authors, however, who have suggested that as many as 75% of patients with an osteoporosis-related vertebral fracture still have pain 1 year after the event (Suzuki et al. 2008, 2009, 2010).

These RCTs have been criticized (Clark et al. 2009). Vertebroplasty aims at internal fixation of non-healed osteoporotic vertebral fractures to reduce pain, similar to that obtained by internal fixation with other acute fractures. However, vertebral fractures usually heal within 8 weeks, whereas the edema found in MRI evaluations persists for an extended period. There is no strict definition regarding time limits when a fracture should be defined as acute, as subacute, or as one with delayed union. The study by Kallmes et al. (2009) involved only outpatients, so that inpatients hospitalized with acute fracture pain were excluded and the protocol required 4 weeks of medical therapy before enrollment was possible. Critics therefore suggest that these enrollment criteria would have resulted in a study on healed fractures where another source of pain should have been considered. According to the critics, a more appropriate selection criterion would have been uncontrolled pain for less than 6 weeks, which was found in only 32% of the subjects in the study reported by Buchbinder et al. (2009) and in 44% of the subjects reported by Kallmes et al. (2009). The trial by Buchbinder et al. was planned to involve 200 patients but only 78 were actually included over 4 years in the final study; this was because 2 of the 4 study hospitals withdrew after including only 5 patients each. As a result, 68% of the procedures were performed in one hospital by one radiologist. Finally, 64% of eligible patients in the Australian study and 70% in the US study declined to participate. All these concerns raise questions regarding patient selection and generalization of inferences.

Some critics have also suggested that the study design was inappropriate, as not all patients with a vertebral fracture benefit from a vertebroplasty (Clark et al. 2009). In many studies, patients with maximal back pain tend to have the greatest improvement in pain score after vertebroplasty—more so than those with a lower degree of pain—and there has been concern that the level of pain in the patients in the RCTs cited was lower than in most patients with vertebral fractures. Enrollment of only those patients with the most severe pain might, however, lead to exaggeration of treatment efficacy due to regression toward the mean effect. One could also question whether patients who receive an injection of an anesthetic should be regarded as unbiased controls, since local anesthetic can have effects for a period exceeding the pharmacological activity of the drug. The larger crossover rates reported by Kallmes et al. (2009) that occurred in the control group than in the treatment group could possibly indicate dissatisfaction with the sham procedure that was not captured by pain scales. But as nearly all crossovers occurred after 1 month and the primary outcome was evaluated after 1 month, this should not have affected the primary outcome evaluation.

The efficacy of balloon kyphoplasty in the treatment of vertebral osteoporotic fractures has now also been evaluated in one multicenter RCT involving 149 kyphoplasty-treated patients and 151 non-surgically treated patients with 1–3 acute vertebral fractures (Wardlaw et al. 2009). The primary outcome was changes from baseline to 1 month, as assessed by SF-36 and at 12 months in quality-of-life evaluation. Both groups improved in terms of quality of life, the kyphoplasty group by an average of 7.2 points (95% CI: 5.7–8.8) and the group that did not undergo surgery by an average of 2.0 points (95% CI: 0.4–3.6), a group difference of 5.2 points on average (95% CI: 2.9–7.4). The authors then inferred that balloon kyphoplasty is an effective procedure for patients with acute vertebral fractures. However, substantial improvement was found in both the operated group and in the group that was not operated. Even though there was a group difference in the outcome score, it can be debated whether the difference was of clinical relevance. In addition, during the 1-year follow-up, 21 of the participants in the kyphoplasty group (14%) had new vertebral fractures, in several cases causing a need for new surgical intervention.

There have also been very few studies directly comparing percutaneous balloon kyphoplasty with percutaneous vertebroplasty. This ought to be done, as there could conceivably be advantages to using the kyphoplasty method since it aims to restore the normal anatomy of the fractured vertebra to reduce the kyphosis in the injured region. By inflating a balloon in the fractured vertebral body, kyphoplasty seeks to recreate the original shape of the compressed vertebral body before the cement fixation. However, it has so far been difficult to show any clinical advantages that justify the higher cost of kyphoplasty relative to vertebroplasty. Recently, both methods were compared in a randomized controlled trial that included 100 patients, where the authors reported that the clinical outcome after 6 months was similar in both treatment groups (Liu et al. 2010).

It is not only important to evaluate whether there is a difference in clinical outcome between surgical and non-surgical treatment, however. All new methods should also be assessed in terms of cost in relation to quality of life. The term cost per QUALY is often used, a term that includes cost per gain in quality-adjusted years of life. There has been a publication dealing with this question, mostly based on the assumption that reported short-term effects achieved with vertebroplasty and kyphoplasty remain in the long term (Wardlaw et al. 2009). Using “willingness to pay” (WTP) of 60,000 euros, some of the theoretical models used in the study suggested that the methods could be cost-effective (Strom et al. 2008). However, there is a need for more studies—also with long-term follow-up and including both clinical outcome and costs to society—before we can draw stricter conclusions about the cost-effectiveness of the methods.

Generally speaking, we should discontinue a treatment when there is convincing evidence that it provides little or no effect. The reported RCTs on vertebroplasty (Gray et al. 2007, Buchbinder et al. 2009, Kallmes et al. 2009, Rousing et al. 2009) provide the best evidence we have to date regarding the efficacy of this method for the treatment of osteoporotic vertebral compression fractures. The repeated findings of improvement in pain in both intervention and control groups and the lack of benefit in the intervention group relative to the control group seems to reflect the benign natural history of pain development in vertebral compression fractures in the elderly. It must then be regarded as questionable to offer patients an intervention that is no more effective, is more expensive, and sometimes more dangerous than placebo (Buchbinder et al. 2008). Most patients, when receiving this information, will probably choose a medical and less risky treatment of the pain. The results of the RCTs should once more remind us to be cautious in using treatments based on data with a low level of evidence. Case reports and observational studies are often biased toward overestimating treatment benefits. Thus, vertebroplasty appears to confer no benefit over a sham procedure or medical care, and it involves adverse risks associated with the operation. In contrast to this, one RCT has shown a statistically significant improvement in the quality of life score after 1 month in a group of patients operated with balloon kyphoplasty in comparison with a group subjected to non-surgical care (Wardlaw et al. 2009). Whether this difference represents not only a statistically significant difference but also a clinically relevant difference should be evaluated in future studies.

In conclusion, lack of evidence of efficacy when advocating vertebroplasty in the treatment of osteporotic vertebral fractures is stronger than the proof of efficacy when using kyphoplasty. The results of published RCTs should lead to the discontinuation of vertebroplasty as a general treatment modality for vertebral compression fractures with persistent pain in the elderly osteoporotic patient, while balloon kyphoplasty ought to be evaluated in further studies before definite recommendations can be given.
